# Emerging role of ARHGAP29 in melanoma cell phenotype switching

**DOI:** 10.1002/1878-0261.70114

**Published:** 2025-09-04

**Authors:** Beatrice Charlotte Tröster, Melanie Kappelmann‐Fenzl, Anja Katrin Bosserhoff, Nicole Rachinger

**Affiliations:** ^1^ Institute of Biochemistry, Friedrich‐Alexander‐University Erlangen‐Nürnberg (FAU) Erlangen Germany; ^2^ Faculty of Computer Science Deggendorf Institute of Technology Deggendorf Germany; ^3^ Comprehensive Cancer Center Alliance WERA (CCC WERA) Erlangen Germany; ^4^ Bavarian Cancer Research Center (BZKF) Erlangen Germany

**Keywords:** ARHGAP29, malignant melanoma, mesenchymal‐like phenotype, phenotype switch, RhoA/ROCK signaling, SMAD signaling

## Abstract

Rho GTPase‐activating protein 29 (ARHGAP29) is an inhibitor of the Ras homolog family member A (RhoA)/Rho‐associated protein kinase (ROCK) signaling pathway. Studies in non‐melanoma cancer entities described that ARHGAP29 modulates the actin cytoskeleton, promoting tumor cell invasion. In melanoma, its function has been completely unknown. Our transcriptomic analyses revealed a strong expression of ARHGAP29 in melanoma cell lines compared to melanocytes. Therefore, we hypothesized that ARHGAP29 affects the migratory potential of melanoma cells and drives melanoma progression. By knocking down ARHGAP29, we demonstrated that it promotes a spread cell morphology through regulating the RhoA/ROCK pathway. Further investigations indicated the role of ARHGAP29 on SMAD activity. Interestingly, our data showed that ARHGAP29 expression is promoting tumor cell plasticity through a mesenchymal‐like, invasive phenotype. To summarize, this study gives insights into the functional role of ARHGAP29 and its downstream signaling in melanoma. Our findings provided evidence supporting the hypothesis that ARHGAP29 is an important player in melanoma progression, a promising and novel target in melanoma treatment.

## Graphical abstract

This study gives first insights into the role of ARHGAP29 in malignant melanoma. ARHGAP29 was revealed to be connected to tumor cell plasticity, promoting a mesenchymal‐like, invasive phenotype and driving tumor progression. Further, it modulates cell spreading by influencing RhoA/ROCK signaling and affects SMAD2 activity.

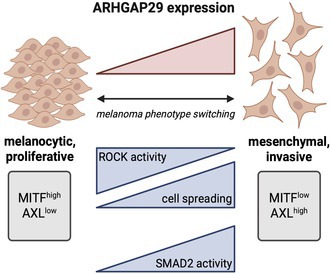

**Abbreviations**
AKT: AKT serine/threonine kinase 1
ARHGAP29: Rho GTPase‐activating protein 29
AXL: AXL receptor tyrosine kinase
BSA: bovine serum albumin
CAM: cell adhesion molecule
CTGF: connective tissue growth factor
ECM: extracellular matrix
ERK1/2: mitogen‐activated protein kinase 1/3
FCS: fetal calf serum
FDR: false‐discovery rate
GAP: GTPase‐activating protein
HRP: horseradish peroxidase
ID1: inhibitor of DNA binding 1
ITGB3: integrin beta 3
LIMK: LIM domain kinase
LUC: Luciferase
MCAM: melanoma cell adhesion molecule
MITF: melanocyte‐inducing transcription factor
MMP: matrix metalloprotease
NHEM: normal human epidermal melanocyte
ns: not significant
PARG1: PTPL1‐associated RhoGAP 1
qRT‐PCR: quantitative Real‐Time PCR
Radil: Ras‐association and dilute domain‐containing protein
Rasip1: Ras interacting protein 1
RhoA: Ras homolog family member A
RIPA: radioimmunoprecipitation assay
ROCK: Rho‐associated protein kinase
scRNA‐Seq: Single‐cell RNA sequencing
SEM: standard error of the mean
siARHGAP29: siPool for ARHGAP29
siCtr: siPool Control
siPool: short interfering RNA pool
SKI: SKI proto‐oncogene
SNAIL: Snail family transcriptional repressor 1
SnoN: SKI like proto‐oncogene
SRC: SRC proto‐oncogene
TBS‐T: tris‐buffered saline with Tween20
TGFBR1: TGFβ receptor 1
TGFBR2: TGFβ receptor 2
TGFβ: transforming growth factor beta

## 1 Introduction

Malignant melanoma is the deadliest form of skin cancer with increasing incidence worldwide. It develops by the malignant transformation of melanocytes, the pigment‐producing cells mainly found in the epidermis [1, 2, 3]. Despite the advanced treatment strategies, the toxicity and limited clinical tolerance of current treatment options still pose a big challenge [4]. Furthermore, as the high metastatic potential of melanoma cells and the development of resistance further complicate the treatment of melanoma [5, 6], there is an urgent need for new therapeutic targets. Another challenge of melanoma treatment is the high heterogeneity of melanoma. Melanoma cells display a variety of phenotypes that differ in their transcriptional profile and therapy response. Generally, two distinct cell states, the proliferative and invasive cell states, can be defined. Melanoma cells can switch between these cell states, which is referred to as phenotype switching [7, 8]. This phenotypic plasticity is discussed to be responsible for the development of resistance. Whereas the proliferative cell state shows better therapy response, the invasive, mesenchymal‐like phenotype is likely to be resistant to treatment [9]. Hence, an important goal is the development of therapy options that target cells displaying the mesenchymal‐like gene signature to prevent the acquirement of resistance and consequent relapse.

Rho GTPases are important regulators of various cellular processes such as cell motility, morphology, cytoskeletal organization, and gene expression. By switching between an inactive GDP‐bound and an active GTP‐bound state, Rho GTPases function as molecular switches, either activating or inactivating their downstream signaling cascades. Therefore, they are critical for the development and progression of cancer and other pathologies [10]. One important Rho GTPase is the Ras homolog family member A (RhoA). While RhoA acts as a driver of tumor development in various types of cancer [11, 12, 13], studies in melanoma have been conflicting. Kaczorowski *et al*. found a correlation between an increased RhoA expression and a prolonged recurrence‐free survival, suggesting a tumor suppressive role of RhoA in melanoma [14]. Another study demonstrated that the inhibition of RhoA/Rho‐associated protein kinase (ROCK) leads to an increase in the invasive capacity of human melanoma cells [15]. Despite that, the knowledge on RhoA in melanoma, including its downstream signaling and molecular functions, is currently still very limited.

The GTPase‐activating protein (GAP) Rho GTPase‐activating protein 29 (ARHGAP29) is an intracellular protein preferably interacting with RhoA [16], and is expressed in various tissues [17]. ARHGAP29, also referred to as PTPL1‐associated RhoGAP 1 (PARG1), suppresses the activity of the RhoA/ROCK signaling pathway by activating the GTPase activity of RhoA [18, 19]. Physiologically, ARHGAP29 plays a role in blood vessel formation by increasing lumen size and the adhesion of endothelial cells to the extracellular matrix (ECM) via integrins [20, 21]. Furthermore, the interaction of ARHGAP29 with Rap1, Ras interacting protein 1 (Rasip1) and Ras‐association and dilute domain‐containing protein (Radil) in endothelial cells promotes cell spreading and influences the endothelial barrier function [22]. In the context of cancer, previous studies have shown that ARHGAP29 reduces the formation of stress fibers via its influence on the RhoA/LIM domain kinase (LIMK)/cofilin pathway, leading to a decrease in cell contractility [23]. Furthermore, it was observed that a high expression of ARHGAP29 correlates with worse survival of patients with renal cell carcinoma, prostate cancer, and breast cancer [17, 18, 24]. Due to these findings, ARHGAP29 is emerging as a potential prognostic marker and target for cancer treatment. However, as of now, the function of ARHGAP29 has not been investigated in malignant melanoma. Hence, we examined the role of ARHGAP29 in human melanoma cell lines, focusing on the effects of ARHGAP29 on tumor cell morphology and its functional effects. We hypothesized that ARHGAP29 would enhance the migration and invasion rate of melanoma cells and would therefore play a role in melanoma progression. We further addressed the involvement of the RhoA/ROCK pathway in the observed effects. Our results give first and novel insights into the role of ARHGAP29 in malignant melanoma and the involved downstream signaling cascades.

## 2 Methods

### 2.1 Cell culture

The experiments were conducted with human melanoma cell lines. Expression analyses were performed with the cell lines *SbCl2* (RRID:CVCL_D732), *WM1158* (RRID:CVCL_6785), *WM1366* (RRID:CVCL_6789), *WM3211* (RRID:CVCL_6797), *WM793* (RRID:CVCL_8787) (generously gifted from Dr. Meenhard Herlyn, Wistar Institute, Philadelphia, PA, USA), *Mel Juso* (RRID:CVCL_1403) and *Mel Im* (RRID:CVCL_1403) were provided by Dr. Judith Johnson (LMU, Munich, Germany), and *SKMel28* (RRID:CVCL_0526) cells obtained from the American Type Culture Collection (ATCC, Virginia, VA, USA) (Table S1), and normal human epidermal melanocytes (NHEM, PromoCell, Heidelberg, Germany). Wistar cells were cultured in a culture medium consisting of MCDB153 (PAN‐Biotech GmbH, Aidenbach, Germany), 20% Leibovitz's L‐15 (PAN‐Biotech GmbH), 2% fetal calf serum (FCS), 1.68 mm CaCl_2_ (Sigma‐Aldrich, Darmstadt, Germany), and 5 μg·mL^−1^ insulin (PAN‐Biotech GmbH, Aidenbach, Germany) in 5% CO_2_ at 37 °C as previously described [25]. Melanoma cell lines *Mel Juso*, *Mel Im*, and *SKMel28*, used for RNA sequencing and plasticity analysis, were cultured as described previously [26]. NHEMs were cultured in melanocyte growth medium M2 (M2 with Supplementary Mix, PromoCell, Heidelberg, Germany) in 5% CO_2_ at 37 °C. The melanoma cells were passaged three times (*WM1158* 1:5; *WM1366* 1:4) and NHEMs once per week via trypsinization. All used cell lines have been authenticated in the past three years, determined by STR‐Profiling technology, and all assays were performed with mycoplasma‐free cells (testing is carried out regularly).

### 2.2 Tissue samples

Human tissue samples (collected in the timeframe between 2012 and 2013) were obtained from the tissue collection of the Institute of Pathology, University of Regensburg, Germany. Sampling and handling of patient material were carried out in accordance with the ethical principles of the Declaration of Helsinki. The use of human tissue material was approved by the local ethics committee of the University of Regensburg (Application Number: 09/11 and 03/151). The experiments were undertaken with the understanding and written consent of each subject. For staining, the anti‐ARHGAP29 antibody (1:100, NBP1‐05989, Novus Biologicals, Littleton, CO, USA) established by the use of siPool treatment (Fig. S1A,B) was used.

### 2.3 Transient transfection

*WM1366* and *WM1158* were seeded into 6‐well plates in a concentration of 1.5–3.0 × 10^5^ cells/well and transfected with an siPool (pool containing approximately 30 specific siRNAs [27]) targeting specifically ARHGAP29 (siARHGAP29, siTOOLs Biotech GmbH, Planegg/Martinsried, Germany). For transfection, the Lipofectamine RNAiMax reagent (Thermo Fisher Scientific Inc., Waltham, MA, USA) was used according to the manufacturer's instructions. Cells were transfected for at least 24 h before harvesting. An siPool Control (siCtr) served as a transfection control. Using the Lipofectamine LTX/Plus reagent (Life Technologies, Carlsbad, CA, USA), cells were transfected with a pcDNA3.1‐CTGF plasmid containing the CTGF coding sequence in a pcDNA3.1 backbone (kindly provided by Rudi Fuchshofer [28]) or the corresponding pcDNA3.1 control vector plasmid according to the manufacturer's instructions. Cells were incubated for 24 h before harvesting. The cell diameter and the ratio between longitudinal and transverse diameter were determined for all cells in five pictures per condition for each implementation.

### 2.4 Inhibitor treatment

*WM1366* and *WM1158* cells were treated with the ROCK inhibitor Y‐27632 (Calbiochem, San Diego, CA, USA) at a working concentration of 10 μm for 24 h if not stated otherwise. Treatment started 24 h after transfection of the cells with the siPools. Y‐27632 is used as standard for the investigation of ROCK signaling [29].

### 2.5 RNA isolation and reverse transcription

Total cellular RNA was isolated from cell pellets using the E.Z.N.A. Total RNA Kit (Omega Bio‐Tek, VWR Darmstadt, Germany) according to the manufacturer's instructions. The isolated RNA was measured with a NanoDrop spectrophotometer (Peqlab Biotechnologie GmbH, Erlangen, Germany) and 500 ng were reverse‐transcribed with the SuperScript II Reverse Transcriptase Kit (Thermo Fisher Scientific Inc.) to generate cDNA as previously described [26].

### 2.6 Quantitative real‐time PCR

For quantitative Real‐Time PCR (qRT‐PCR), the LightCycler 480 system (Roche, Mannheim, Germany) was used. The measurement was performed with 1 μL of the cDNA samples and specific primers (Table S2) at a concentration of 20 μm. The annealing temperature was set to 60 °C, and β‐actin was used as the reference gene for normalization.

### 2.7 Protein isolation

Cell pellets were resuspended in 50–150 μL radioimmunoprecipitation assay (RIPA) buffer in order to generate protein lysates and were prepared as previously described [30]. The protein concentration was measured using the Pierce BCA Protein Assay Kit (Thermo Fisher Scientific Inc.) and the CLARIOstar Plus microplate reader (BMG Labtech GmbH, Ortenberg, Germany).

### 2.8 Western blot analysis

As described previously [30], 30 μg of protein lysate per lane was applied onto a 10–12.75% SDS gel for protein separation and blotted onto PVDF membrane (Cytiva Life Sciences, Marlborough, MA, USA). The membrane was blocked with 3–5% bovine serum albumin (BSA) in tris‐buffered saline with Tween20 (TBS‐T). After incubation with the primary antibody (Table S3) at 4 °C overnight, the secondary antibody (Table S3) was added to the membrane for 1 h. The detection of the luminescence signal was performed by using the ECL Plus Western Blotting Detection Kit (Bio‐Rad Laboratories Inc., Hercules, CA, USA) and the Intas ECL Chemocam LabImager (Intas Science Imaging Instruments GmbH, Goettingen, Germany). For the densitometric quantification, the LabImage Software (Kapelan Bio‐Imaging GmbH, Leipzig, Germany) was used.

### 2.9 Immunofluorescence

30 000 cells were seeded onto cover slips in 12‐well plates. After 24 h, fixation with 4% paraformaldehyde/PBS or glyoxal solution, and permeabilization and blocking with 1% BSA/0.1% Triton‐X100/PBS for 30 min followed. Cells were stained with rabbit anti‐phospho‐SMAD2 antibody (1:100 #3108, Cell Signaling, Leiden, the Netherlands) and secondary antibody (1:1000 #A32732, Alexa Fluor 555 anti‐rabbit IgG red fluorescence 555 Invitrogen, Darmstadt, Germany) for 1 h each at room temperature. For analyzing the cytoskeleton, cells were stained for actin with 1:140 Rhodamine Phalloidin (PHDR1, 14 μm, tebu‐bio, Offenbach, Germany) for 45 min. The nucleus was stained with DAPI (0.1 mg·mL^−1^, Merck kGaA, Darmstadt, Germany) for 30 min. Aqua‐Poly/Mount (Polysciences, Warrington, PA, USA) was utilized as the mounting medium. For analyzing the cell surface area, ten cells per condition were analyzed in each implementation (total cell number of 30). For the investigation of pSMAD2, 30 cells were analyzed in total.

### 2.10 Spheroid assay

8000 cells per well were seeded into 96‐well plates, coated by adding 100 μL of a 1% agarose gel to the wells. Y‐27632 (final concentration: 10 μm) was added to the cell suspension right before seeding the cells. Five spheroids per condition were generated, left to grow for 2 days, and evaluated.

### 2.11 Boyden chamber assays

Migration and invasion assays were performed as described previously [31]. The lower chamber of a Boyden chamber (NeuroProbe, Gaithersburg, MD, USA) was filled with 210 μL of fibroblast‐conditioned medium as a chemoattractant. For cell migration, a gelatin‐covered filter membrane and for cell invasion, filter membranes that had been covered with 52 μL of diluted Matrigel (1:3 in serum‐free medium) were used. The upper chamber was filled with 20 000 (migration) or 200 000 (invasion) treated cells resuspended in DMEM without FCS. Each condition was prepared in triplicates for each run. After incubation at 37 °C for 4 h, the filter membranes were fixed and stained with Hemacolor Rapid staining. The average number of cells on 10 fields of view per membrane was determined.

### 2.12 Cell viability assay

The cell number was measured with the Cell Proliferation Kit II (XTT, Roche Diagnostics GmbH) by determining the cellular metabolic activity. 2000 cells per well were seeded in triplicates in 96‐well plates. On the day of measurement, the medium was changed to medium without phenol red (PAN‐Biotech GmbH, Aidenbach, Germany). The measurement of cell viability was performed according to the manufacturer's instructions on days 1 to 4 (day 1 means 24 h after seeding). The absorbance was determined with the CLARIOstar Plus microplate reader (BMG Labtech GmbH, Ortenberg, Germany) at 490 nm after 2 h of incubation.

### 2.13 Clonogenic assay

24 h after transfection, 200 or 500 cells per well were seeded into 6‐well plates and left to grow for 8 days. Staining and fixation were performed with 6% glutaraldehyde and 0.36% crystal violet for 30 min at room temperature. The number and size of the colonies were determined. The analysis was performed three times in duplicates.

### 2.14 Luciferase assay

The activity of TGFβ and BMP in human *WM1158* and *WM1366* cell lines was determined using luciferase (LUC) assays. The respective cell lines were seeded into 6‐well plates in duplicates and were transiently transfected with an siPool against ARHGAP29 for 24 h as described in 2.3 Transient transfection. Afterwards, cells were treated with ROCK inhibitor Y‐27632 (not for BMP measurements) as described before in 2.4 Inhibitor treatment. After another 24 h of incubation, cells were transiently transfected with plasmid DNA containing CAGA for TGFβ [32] (generous gift from Steve Dooley) and BMP‐responsive element for BMP [33] (Addgene plasmid #45126; http://n2t.net/addgene:45126; RRID: Addgene_45 126) on a pGL3Promotor‐Plasmid or the empty vector, and co‐transfected with a pRL‐TK control vector (Promega Corp., Madison, WI, USA) using Lipofectamine LTX/Plus reagent according to the manufacturer's instructions as described before [34]. Cells were lysed 24 h after transfection, and the firefly LUC activity was quantified by a luminometric assay (Promega Corp.).

### 2.15 RNA sequencing library preparation, mapping, and statistical analysis

RNA sequencing (RNA‐Seq) samples and libraries were prepared as described previously [35]. Pellets of each cell line (*NHEM*, *SbCl2*, *WM3211*, *WM793*, *WM1366*, *WM1158*, *Mel Juso*, *Mel Im*, and *SKMel28*) were taken, followed by RNA isolation and library preparation to reflect an average expression profile of each cell line separately. This was performed with at least two biological replicates. Sequencing was performed according to the paired‐end RNA sequencing protocols from Illumina on a HiSeq2000 with paired‐end module (Illumina, Inc., San Diego, CA, USA). Fifty samples were sequenced from each side of a fragment ~100 bp long with an average number of 20 million reads per sample. Paired‐end reads were aligned to the human reference genome sequence (hg38) using the STAR alignment software (v2.5.2a) [36]. Only reads that mapped to a single unique location were considered for further analysis. Counts for RNA‐Seq reads were calculated using the feature‐counts software (v1.4.6‐p5) [37]. The raw RNA‐Seq counts were used for differential gene expression analysis that was performed using DESeq2 (v1.14.1) [38]. Differentially expressed genes with a false‐discovery rate (FDR) < 0.05 were regarded as statistically significant. Normalization was performed by library size based on the raw counts. Genome Ontology annotation and RNA‐Seq reads annotation were performed using scripts provided by STAR (based on GENCODE v24).

### 2.16 Microscopy

Cells (bright field assays e. g. cell diameter, spheroid assay, Boyden chamber, clonogenic and immunofluorescence) were analyzed with the IX83 fluorescence microscope (Olympus, Hamburg, Germany) and the cellSens Dimension Software 2.3 (Olympus Cooperation).

### 2.17 Statistical analysis

If not otherwise described, each experiment was performed at least three times. The generation of graphs and the statistical analyses were performed with GraphPad Prism 5 (GraphPad Software, Inc., San Diego, CA, USA). The data were analyzed with the Student's *t*‐test if not stated otherwise; the results were depicted as the mean ± standard error of the mean (SEM). A value of *P* < 0.05 was defined as statistically significant. The degree of significance was further specified as follows: ns: not significant, *P < 0.05, **P < 0.01, ***P < 0.001, ****P ≤ 0.0001.

## 3 Results

### 3.1 ARHGAP29 is upregulated in human melanoma cell lines

**Fig. 1:**
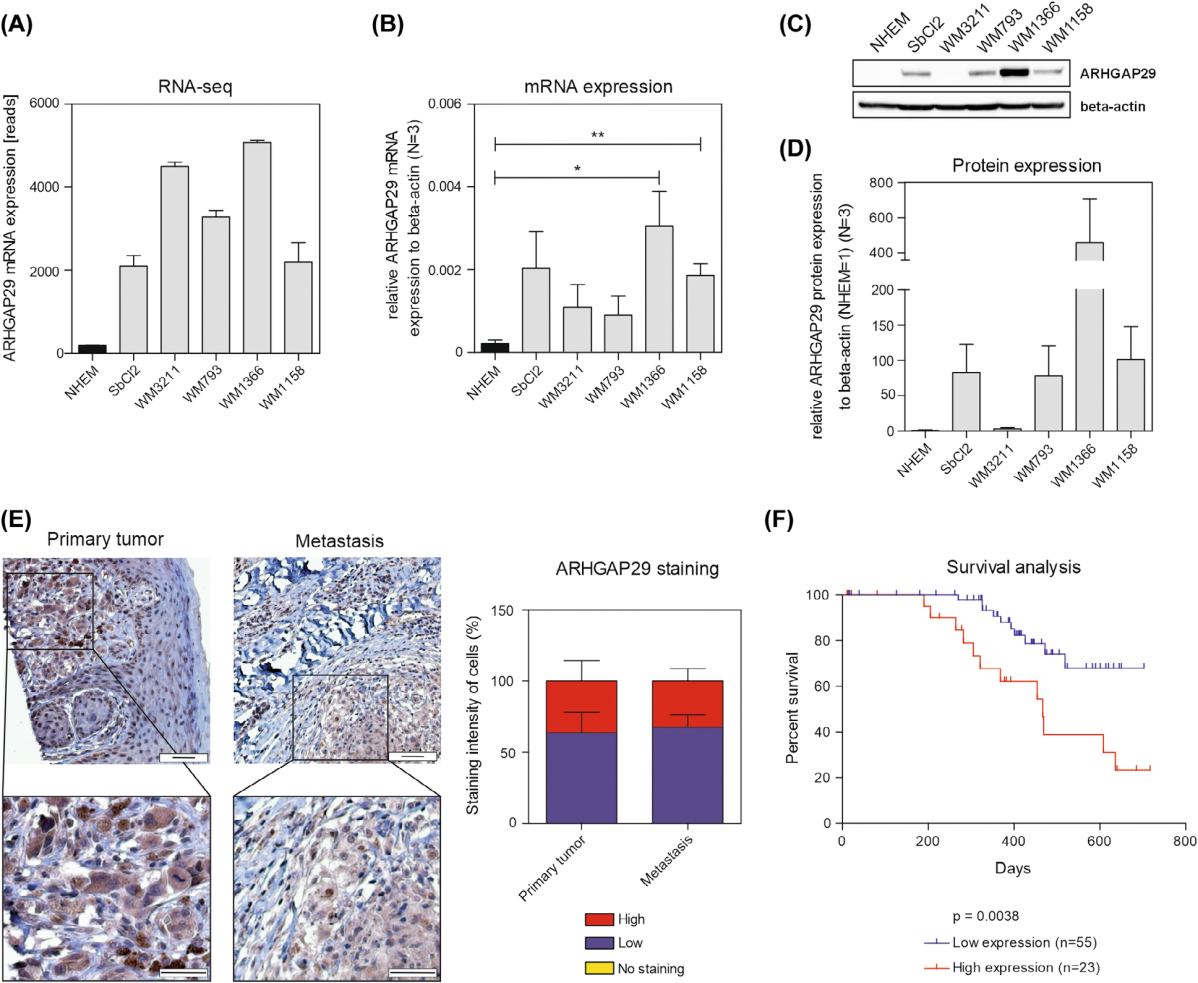
Expression analysis of ARHGAP29 in malignant melanoma. (A) Analysis of RNA sequencing (RNA‐Seq) count data [25] for ARHGAP29 in normal human epidermal melanocytes (NHEMs), and in the melanoma cell lines *SbCl2*, *WM3211*, *WM793*, *WM1366*, and *WM1158*. (B) mRNA expression and (C, D) protein expression of ARHGAP29 measured with the qRT‐PCR system and by western blot, respectively, in the cell lines mentioned in (A) (*N* = 3). The expression level was normalized to β‐Actin. NHEMs were used for normalization of the protein expression (NHEM = 1). (E) Immunohistochemistry in human tissue samples derived from primary melanoma and melanoma metastases depicts the expression of ARHGAP29 in melanoma cells. Cells were counted depending on the staining intensity—high (red), low (blue), and no staining (yellow). “No staining” was not found (*N* = 3) (scale bar: 50 μm). Analysis with column statistics *t*‐test showed compared to no‐staining: low staining: *P* = 0.0189; high staining: *P* = 0.0362. (F) Kaplan–Meier survival curves of melanoma patients based on the expression level of ARHGAP29 (generated with data from The Human Protein Atlas [39]). The curves were compared with the log‐rank test. For all pictures: Data were analyzed with the Student's *t*‐test if not stated otherwise. Depicted is the mean ± standard error of the mean (SEM). *P < 0.05, **P < 0.01.

In order to gain insight into the role of ARHGAP29 in malignant melanoma, we first performed expression analyses. Our RNA‐Seq data [25] showed a highly increased expression of ARHGAP29 in melanoma cell lines compared to normal human epidermal melanocytes (NHEMs) (Fig. 1A). These data were supported by qRT‐PCR and western blot analysis (Fig. 1B–D). Further, staining of human tissue samples showed comparable results. We observed weak (depicted in blue) to high (red) expression in primary tumors and metastasis and, importantly, no sample without staining (Fig. 1E). Survival data [39] showed that a high protein expression of ARHGAP29 in melanoma patients correlates with worse survival, suggesting an important role of ARHGAP29 in melanoma (Fig. 1F).

### 3.2 ARHGAP29 promotes cell spreading in melanoma cells

**Fig. 2:**
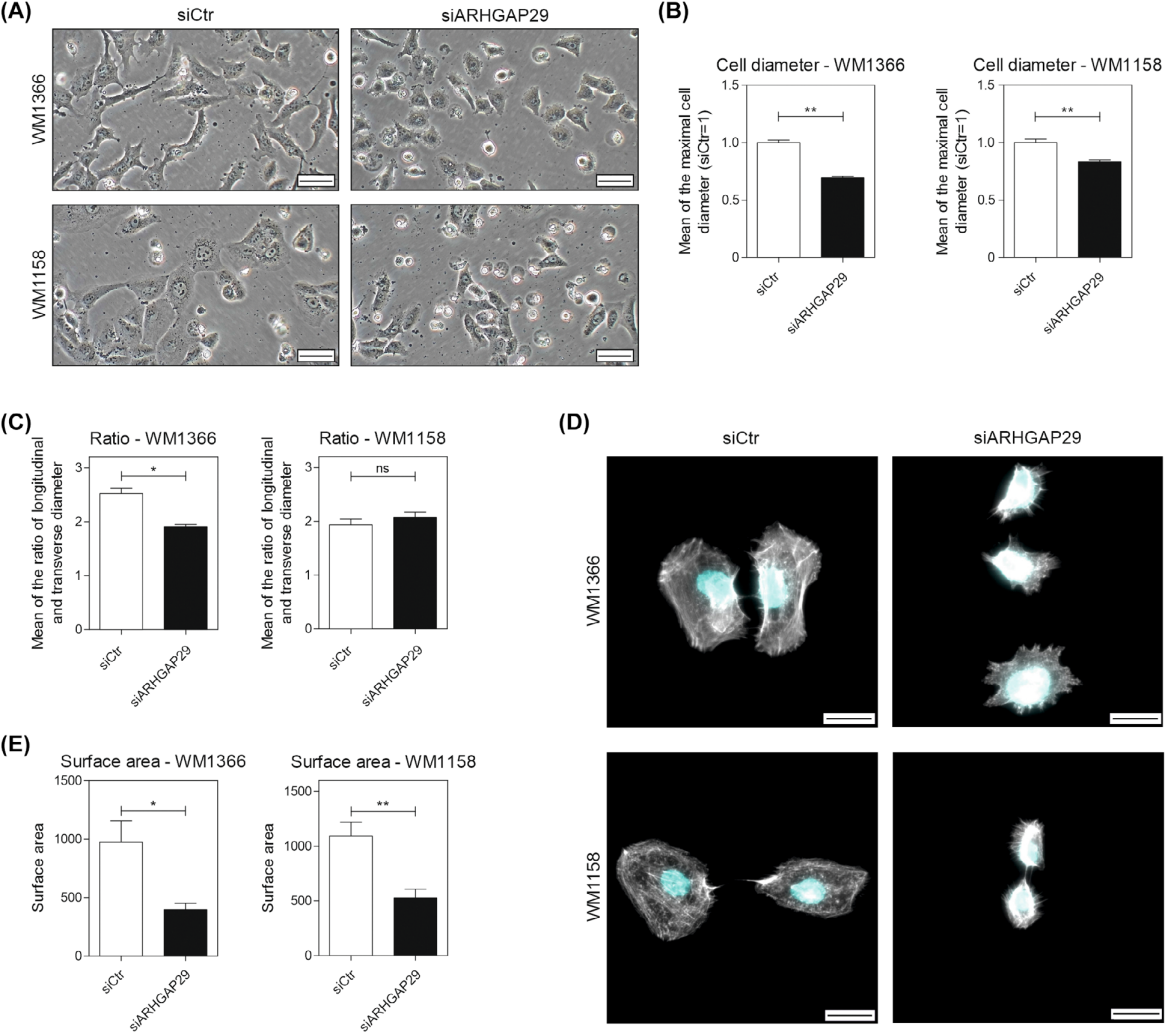
ARHGAP29 promotes the spreading of melanoma cells. (A) Exemplary images of the cell morphology of *WM1366* and *WM1158* cells after the treatment with siARHGAP29 for 48 h (scale bar: 50 μm). (B) The cell diameter of the cells (siCtr = 1) and (C) the ratio between longitudinal and transverse diameter was determined in three replicates. Per replicate, five fields per view were made, and all cells were analyzed (in total, 150–300 cells·view^−1^). (D, E) Cells were stained for the actin cytoskeleton (Phalloidin = white) and the nucleus (DAPI = blue) (scale bar: 20 μm). The surface area of 10 cells per condition was quantified (*N* = 3). Significance determined by Student's *t*‐test. Error bars depict the mean ± SEM. ns, not significant, *P < 0.05, **P < 0.01.

In this study, we selected two melanoma cell lines, *WM1366* and *WM1158*, according to their progression (primary tumor/metastasis) and mutation status (Table S1) to assess the function of ARHGAP29 in melanoma. We established an siPool‐mediated knockdown of ARHGAP29 and revealed a significant reduction in mRNA and protein level (Fig. S1A,B). Upon ARHGAP29 knockdown, we observed a drastic change in cell morphology (Fig. 2A). The cells appeared smaller, less spread, and the maximal cell diameter was significantly reduced (Fig. 2B). In addition, a reduction in the ratio between the longitudinal and transverse cell diameter was measured in *WM1366*, indicating a rounder cell shape after ARHGAP29 knockdown (Fig. 2C). This effect could not be confirmed in *WM1158*, presumably due to the already rounder shape of *WM1158* compared to *WM1366* cells. This may limit the effect of the ARHGAP29 knockdown on cell roundness in *WM1158*. For validation, we stained the cells with phalloidin, a marker for filamentous actin (Fig. 2D). Quantification of the surface area confirmed the reduced spreading of 59% in *WM1366* and 52% in *WM1158* cells after ARHGAP29 knockdown (Fig. 2E) and therefore ARHGAP29's effect on the cytoskeleton and cell morphology in melanoma.

### 3.3 ARHGAP29 enhances tumor‐promoting properties in malignant melanoma

**Fig. 3:**
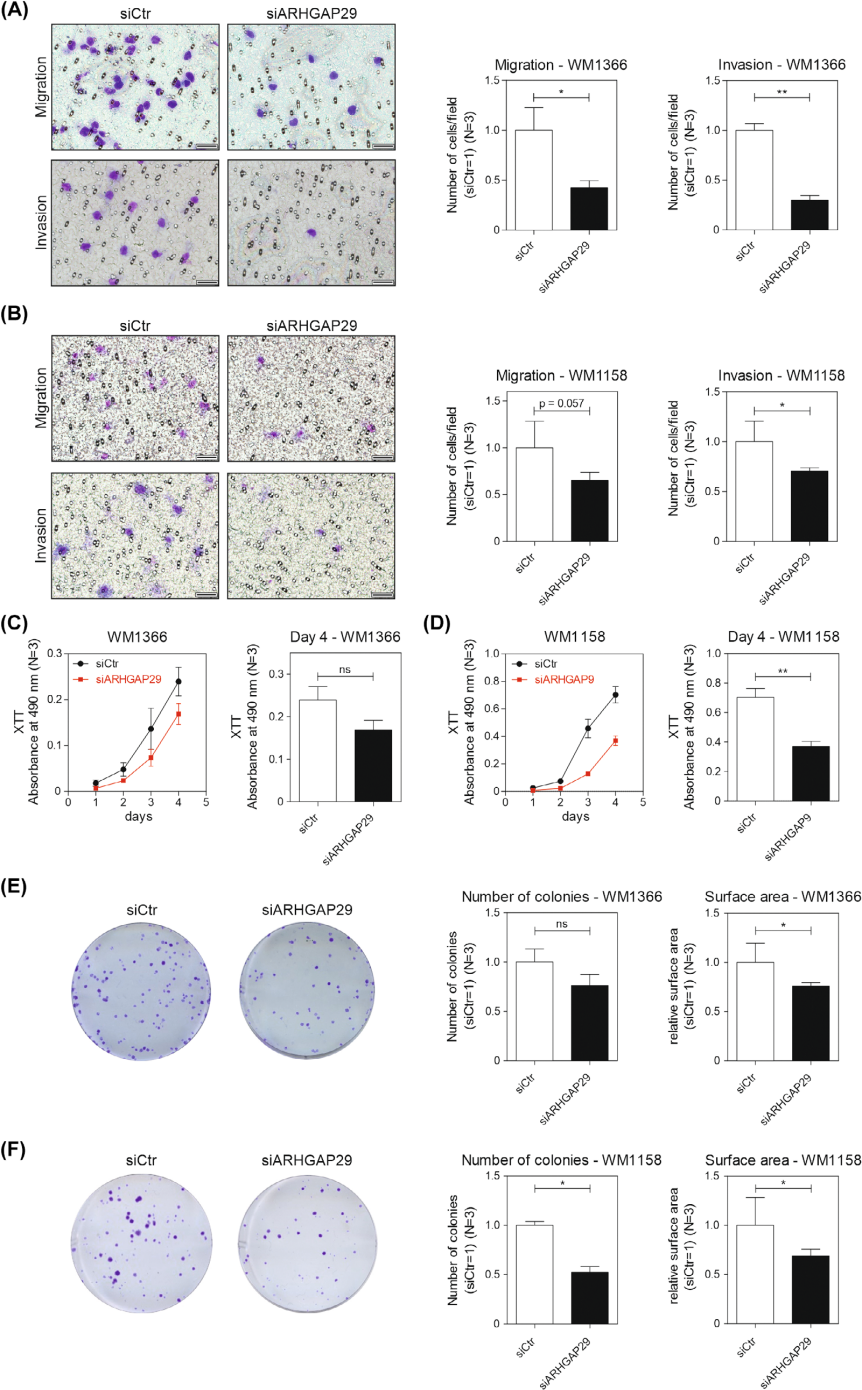
ARHGAP29 stimulates oncogenic properties of melanoma cells. Cell migration and invasion were analyzed with the Boyden chamber assay using cells of the cell lines (A) *WM1366* and (B) *WM1158* after the knockdown of ARHGAP29 (scale bar: 50 μm). The average number of cells per field in the control condition was used for normalization (siCtr = 1) (*N* = 3). Cell number of transfected cells of the cell lines (C) *WM1366* and (D) *WM1158* was measured with the XTT assay (*N* = 3). Colony formation (200 cells·well^−1^) was assessed with siARHGAP29‐transfected cells of the cell lines (E) *WM1366* and (F) *WM1158*. Surface area and colony number were determined, and the data of the control condition were used for normalization (siCtr = 1) (*N* = 3). Significance determined by Student's *t*‐test. Error bars depict the mean ± SEM. ns, not significant, *P < 0.05, **P < 0.01.

Given our prior results, we wondered whether these morphology changes mediated by ARHGAP29 would also have an impact on cell function. Therefore, we investigated the influence of ARHGAP29 on cell migration and invasion with Boyden chamber assays. Cell migration and invasion were reduced after ARHGAP29 knockdown in both cell lines (Fig. 3A,B). We further examined the influence of ARHGAP29 on cell number and colony formation. The cell viability assay revealed a reduced cell number after ARHGAP29 knockdown in *WM1366* (Fig. 3C) and in *WM1158* (Fig. 3D). Besides this finding, the number of colonies as well as the colony size were decreased after siARHGAP29 treatment (Fig. 3E,F), suggesting an influence of ARHGAP29 on the proliferative capability of melanoma cells. Taken together, these results indicate that ARHGAP29 enhances tumor progression by increasing the tumor‐promoting properties, including migration, invasion, and proliferative capability.

### 3.4 ARHGAP29 alters cell–cell adhesion and influences the expression of MCAM

**Fig. 4:**
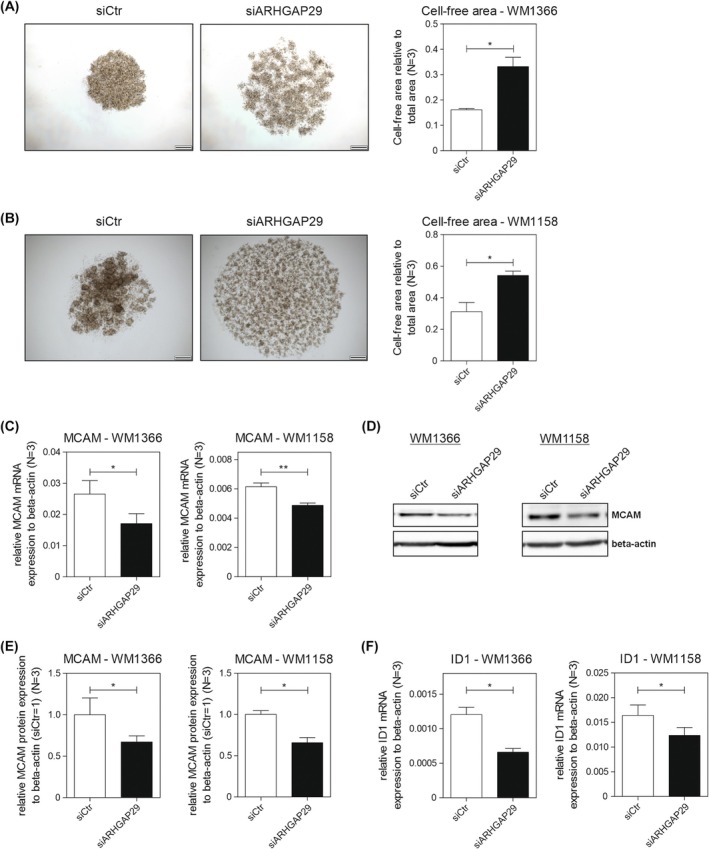
ARHGAP29 knockdown reduces cell–cell adhesion and decreases the expression of MCAM. Spheroids were generated with cells of the cell lines (A) *WM1366* and (B) *WM1158*. The cell‐free area of five spheroids per condition was determined (scale bar: 200 μm) (*N* = 3). The expression of MCAM was determined in both cell lines on (C) mRNA and (D, E) protein level by qRT‐PCR and western blot, respectively (*N* = 3). (F) The expression of ID1 was assessed by qRT‐PCR in both cell lines (*N* = 3). The expression of MCAM and ID1 was normalized to β‐Actin. The protein expression of MCAM in the siCtr‐transfected cells was used for normalization of the protein expression (siCtr = 1). Significance determined by Student's *t*‐test. Error bars depict the mean ± SEM. *P < 0.05, **P < 0.01.

In order to gain further insights into the functional effects of ARHGAP29, we investigated the molecular mechanisms behind the observed effects. We speculated that the effects on cell motility could be due to changes in cell adhesion, and therefore analyzed the spheroid formation after ARHGAP29 knockdown. Interestingly, siARHGAP29 substantially impaired spheroid stability. The spheroids tended to fall apart, resulting in large patches of cell‐free areas (Fig. 4A,B). Due to that, we assumed that ARHGAP29 regulates the expression of cell adhesion molecules (CAMs), thus impairing cell–cell adhesion. Analyses of melanoma cell adhesion molecule (MCAM) demonstrated a significantly reduced expression after siARHGAP29 on mRNA and protein levels (Fig. 4C–E). A loss of homotypic adhesion due to the decreased expression of MCAM explains the instability of the spheroids after siARHGAP29 treatment. Furthermore, investigation of the expression of inhibitor of DNA binding 1 (ID1), a downstream target of MCAM [40], validated our data (Fig. 4F). Overall, ARHGAP29 induces an alteration of the gene expression profile of CAMs that changes the dynamics of cell–cell adhesion, in turn leading to increased cell motility and promoting the formation of distant metastases.

### 3.5 ARHGAP29 regulates the expression of tumor‐promoting genes

**Fig. 5:**
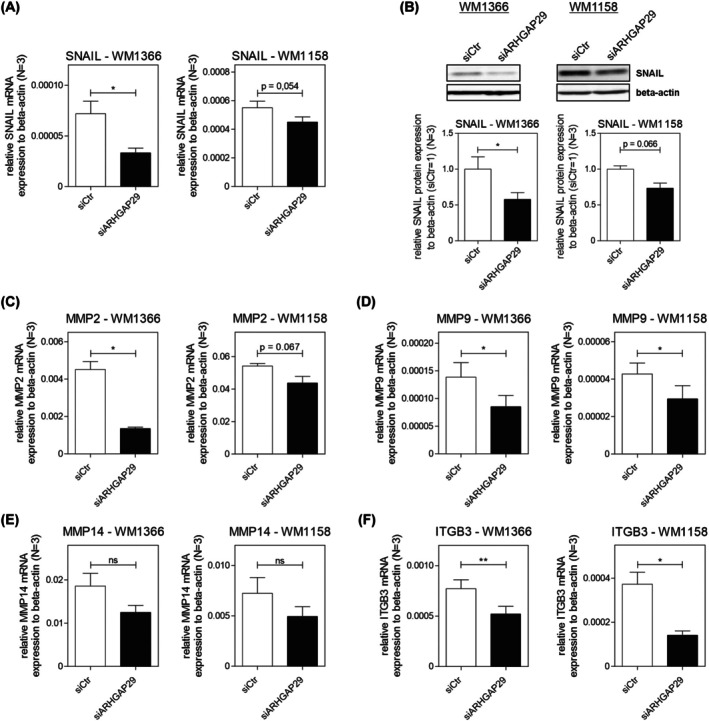
ARHGAP29 knockdown reduces the expression of genes involved in melanoma progression. The expression of SNAIL was investigated in the cell lines *WM1366* and *WM1158* on (A) mRNA and (B) protein levels by qRT‐PCR and western blot, respectively (*N* = 3). The expression of (C) MMP2, (D) MMP9, (E) MMP14, and (F) ITGB3 was analyzed by qRT‐PCR in both cell lines (*N* = 3). The expression levels of the investigated genes were normalized to β‐Actin. The protein expression of SNAIL in the siCtr‐transfected cells was used for normalization of the protein expression (siCtr = 1). Significance determined by Student's *t*‐test. Error bars depict the mean ± SEM. ns, not significant, *P < 0.05, **P < 0.01.

After our results revealed a regulation of MCAM expression, we assessed the influence of ARHGAP29 on different tumor‐promoting genes. We discovered that ARHGAP29 increased the expression of Snail family transcriptional repressor 1 (SNAIL) (Fig. 5A,B) and further detected a reduced expression of different matrix metalloproteases (MMPs: MMP2, MMP9, and MMP14) after ARHGAP29 knockdown (Fig. 5C–E). Particularly, the expression of these genes was highly affected in *WM1366* a tendency was observed in *WM1158*. As MMPs degrade the extracellular matrix (ECM), expose integrin‐ECM binding sites, and are therefore heavily involved in the migration and invasion of melanoma cells [41, 42], a reduced expression of MMPs explains the effects of ARHGAP29 on melanoma cell motility (Fig. 3A,B). As the effects of ARHGAP29 on the expression of, particularly, SNAIL and MMP2 were markedly lower in *WM1158*, it is not surprising that the influence of ARHGAP29 on cell motility was also less profound in this cell line (Fig. 3B). Besides this, we wanted to explore ARHGAP29's effect on the cell‐matrix adhesion between the melanoma cells and the ECM, as this could further impact the movement of cells through the tissue. Previous research has shown that integrin beta 3 (ITGB3) increases the invasive potential of melanoma cells [43]. Therefore, we analyzed whether there is an influence of ARHGAP29 on ITGB3 and detected a significantly decreased expression of ITGB3 after ARHGAP29 knockdown (Fig. 5F). Taken together, the combined increase in the expression of MCAM, SNAIL, ITGB3, and MMPs driven by ARHGAP29 strongly proposes that ARHGAP29 supports the movement of melanoma cells through the ECM, which increases metastasis formation and thereby promotes tumor progression.

### 3.6 ARHGAP29 can signal independently of the RhoA/ROCK pathway

**Fig. 6:**
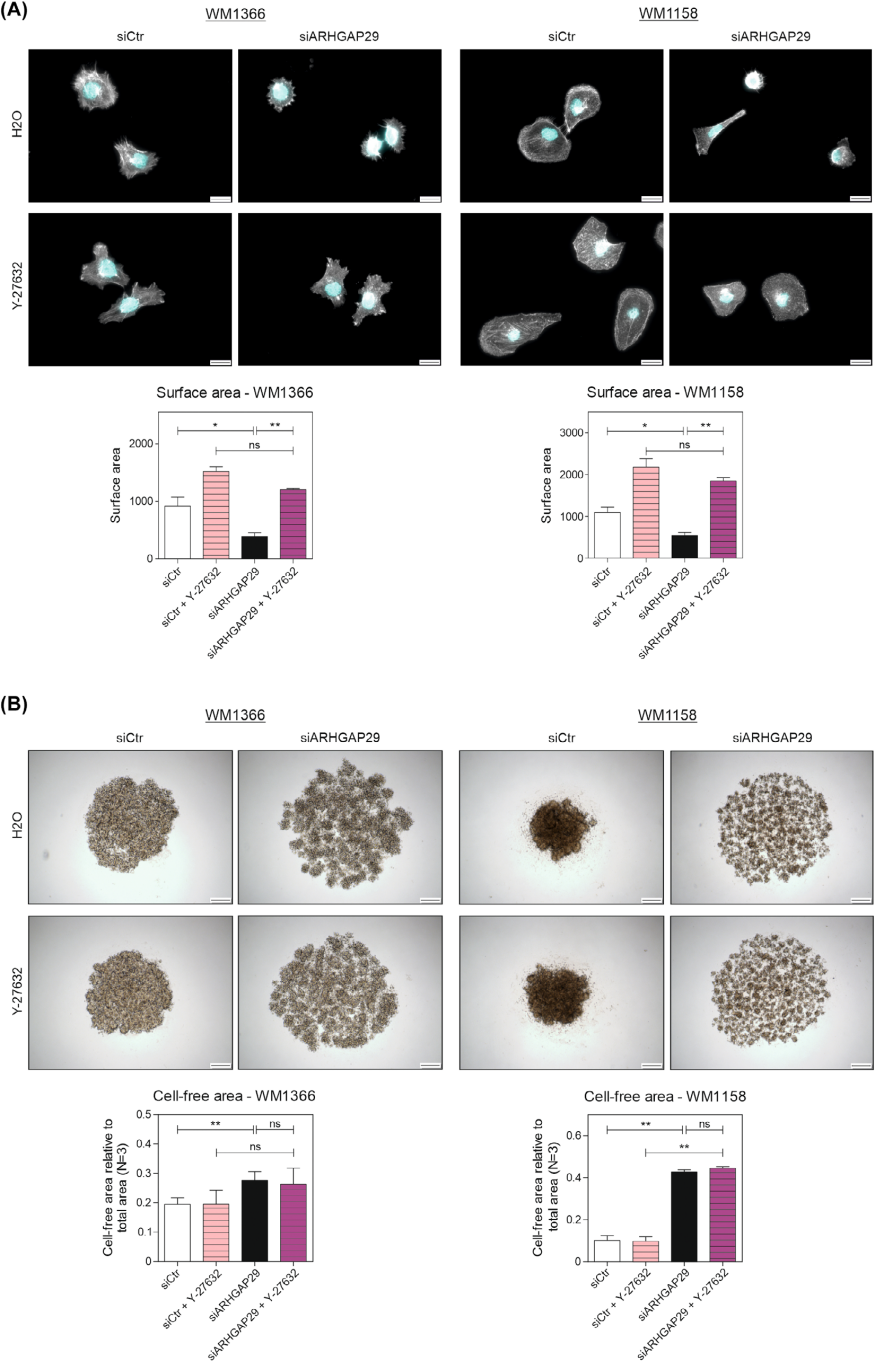
ARHGAP29 affects melanoma cells independently of its inhibitory influence on ROCK. After the knockdown of ARHGAP29, cells of the cell lines *WM1366* and *WM1158* were treated with 10 μm of the ROCK inhibitor Y‐27632. (A) Cells were stained for the actin cytoskeleton (Phalloidin = white) and the nucleus (DAPI = blue) (scale bar: 20 μm). The surface area of 10 cells per condition was determined in three replicates (*N* = 3). (B) Spheroids were generated and treated with the ROCK inhibitor. The cell‐free area of five spheroids per condition was determined (scale bar: 200 μm) (*N* = 3). Significance determined by Student's *t*‐test. Error bars depict the mean ± SEM. ns, not significant, *P < 0.05, **P < 0.01.

Next, we investigated the signaling pathways affected by ARHGAP29. Because ARHGAP29 is an inhibitor of the RhoA/ROCK signaling pathway, we analyzed whether the observed effects on melanoma cells were caused by the regulatory function of ARHGAP29 on the RhoA/ROCK pathway. Therefore, we treated the cells with a well‐known ROCK inhibitor (Y‐27632) after siARHGAP29 treatment and expected the inhibitor to mimic the impact of ARHGAP29 on the tumor cells and reverse the effects of the ARHGAP29 knockdown. As depicted in Fig. 6A, the surface area of siARHGAP29‐treated cells increased significantly after Y‐27632 treatment, and the significant difference in cell spreading was abolished. Thus, the inhibition of ROCK rescued the effect of the ARHGAP29 knockdown on cell morphology as expected. Surprisingly, however, the inhibition of ROCK was not capable of rescuing the effect of ARHGAP29 knockdown on the stability of the spheroids (Fig. 6B). Regarding the regulated tumor‐promoting genes (MCAM, SNAIL, MMP2, ITGB3), the ROCK inhibitor could not rescue the effect of the ARHGAP29 knockdown (Fig. S2A,B). This intriguing finding strongly implies that ARHGAP29 not only signals solely through the RhoA/ROCK pathway but also affects the cells independently of its influence on ROCK.

### 3.7 ARHGAP29 stimulates the SMAD signaling pathway

**Fig. 7:**
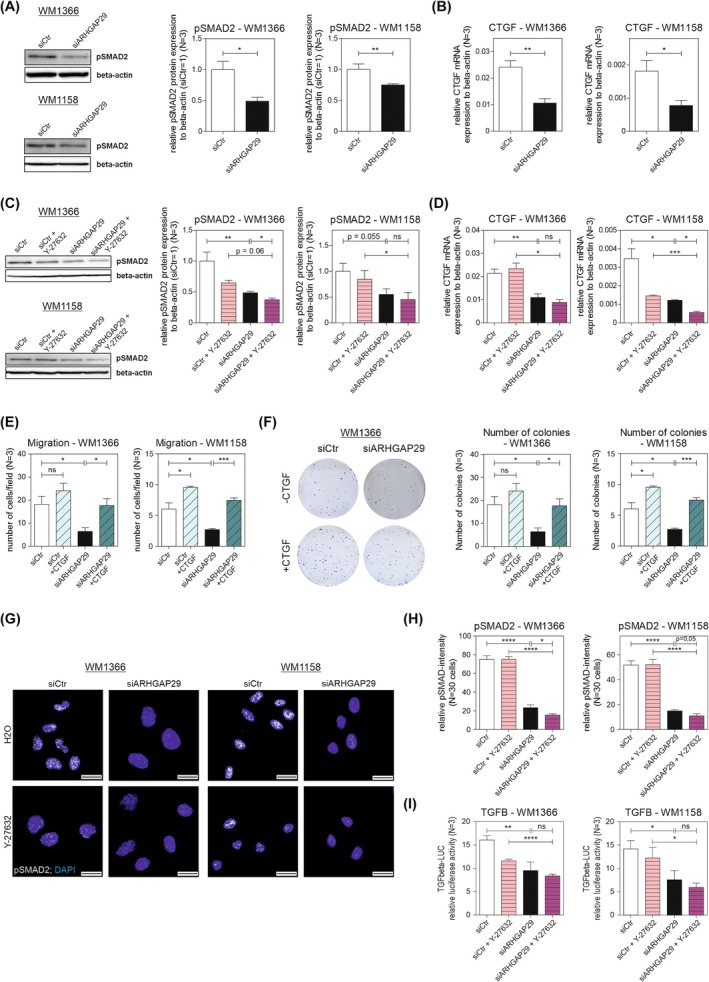
ARHGAP29 stimulates the SMAD signaling pathway independently of its inhibitory influence on ROCK. The protein levels of pSMAD2 (A) and the mRNA expression of CTGF (B) were investigated in the cell lines *WM1366* and *WM1158* by western blot and qRT‐PCR, respectively (*N* = 3). Cells were treated with 10 μm of Y‐27632 and the protein levels of pSMAD2 (C) and the mRNA expression of CTGF (D) in the cell lines *WM1366* and *WM1158* were assessed by western blot and qRT‐PCR, respectively (*N* = 3). The expression levels of CTGF and protein levels of pSMAD2 were normalized to β‐Actin. The protein level of pSMAD2 in siCtr‐transfected cells was used for normalization (siCtr = 1). (E) Migration assay and (F) clonogenic assay (500 cells·well^−1^) of *WM1366* and *WM1158* treated with siARHGAP29 and CTGF overexpression (*N* = 3). (G) Immunofluorescence staining of pSMAD2 (white) and DAPI (blue) in *WM1366* and *WM1158* containing ARHGAP29 knockdown and ROCK inhibitor treatment (scale bars: 20 μm). (H) Quantitative analysis of pSMAD2 intensity of the depicted cells in (G). For quantification, 30 cells per condition were analyzed (*N* = 30). (I) Luciferase assay to assess the TGFβ activity in siARHGAP29 and Y‐27632 treated cells (*N* = 3). Significance determined by Student's *t*‐test. Error bars depict the mean ± SEM. ns, not significant, *P < 0.05, **P < 0.01, ***P < 0.001, ****P ≤ 0.0001.

To unravel the RhoA/ROCK‐independent pathway of ARHGAP29 signaling, we investigated ARHGAP29's influence on the activity of other signaling pathways. We did not identify changes in the activity of AKT serine/threonine kinase 1 (AKT) (Fig. S3A) and mitogen‐activated protein kinase 1/3 (ERK1/2) (Fig. S3B). We further investigated the transforming growth factor beta (TGFβ) pathway but neither discovered differences in the expression levels of TGFβ1 and TGFβ3 nor of the TGFβ receptor 1 (TGFBR1) and 2 (TGFBR2) (Fig. S3C) nor on BMP signaling (Fig. S4A,B). However, we found reduced pSMAD2 levels after siARHGAP29 treatment as well as a decreased expression of the SMAD target gene connective tissue growth factor (CTGF) (Fig. 7A,B). Y‐27632 treatment enhanced the effects of ARHGAP29 knockdown on pSMAD2 levels and CTGF expression (Fig. 7C,D). For validation, overexpression of CTGF was performed. Migration assay (Fig. 7E) and clonogenic assay (Fig. 7F) showed a rescue of the functional effects mediated by ARHGAP29 after CTGF re‐expression. Together, these findings indicate that ARHGAP29 regulates SMAD activity, which can be partially influenced by ROCK. To further investigate the effect of the ROCK inhibitor on pSMAD2, immunofluorescence staining of ARHGAP29 knockdown cells treated with the ROCK inhibitor was performed (Fig. 7G). Quantitative analysis showed a reduced pSMAD2 intensity after ARHGAP29 knockdown that was additionally reduced upon Y‐27632 treatment (Fig. 7H). Investigation of TGFβ showed no additional significant effect of ROCK inhibitor treatment on TGFβ activity after siARHGAP29 treatment (Fig. 7I). So far, we demonstrated that ARHGAP29 not only signals through ROCK, consequently leading to changes in cell morphology, but also induces SMAD signaling that is partially affected by ROCK and possibly affects the expression of various SMAD target genes that increase metastasis formation and drive melanoma progression.

### 3.8 ARHGAP29 expression is increased in mesenchymal‐like, invasive melanoma cells

**Fig. 8:**
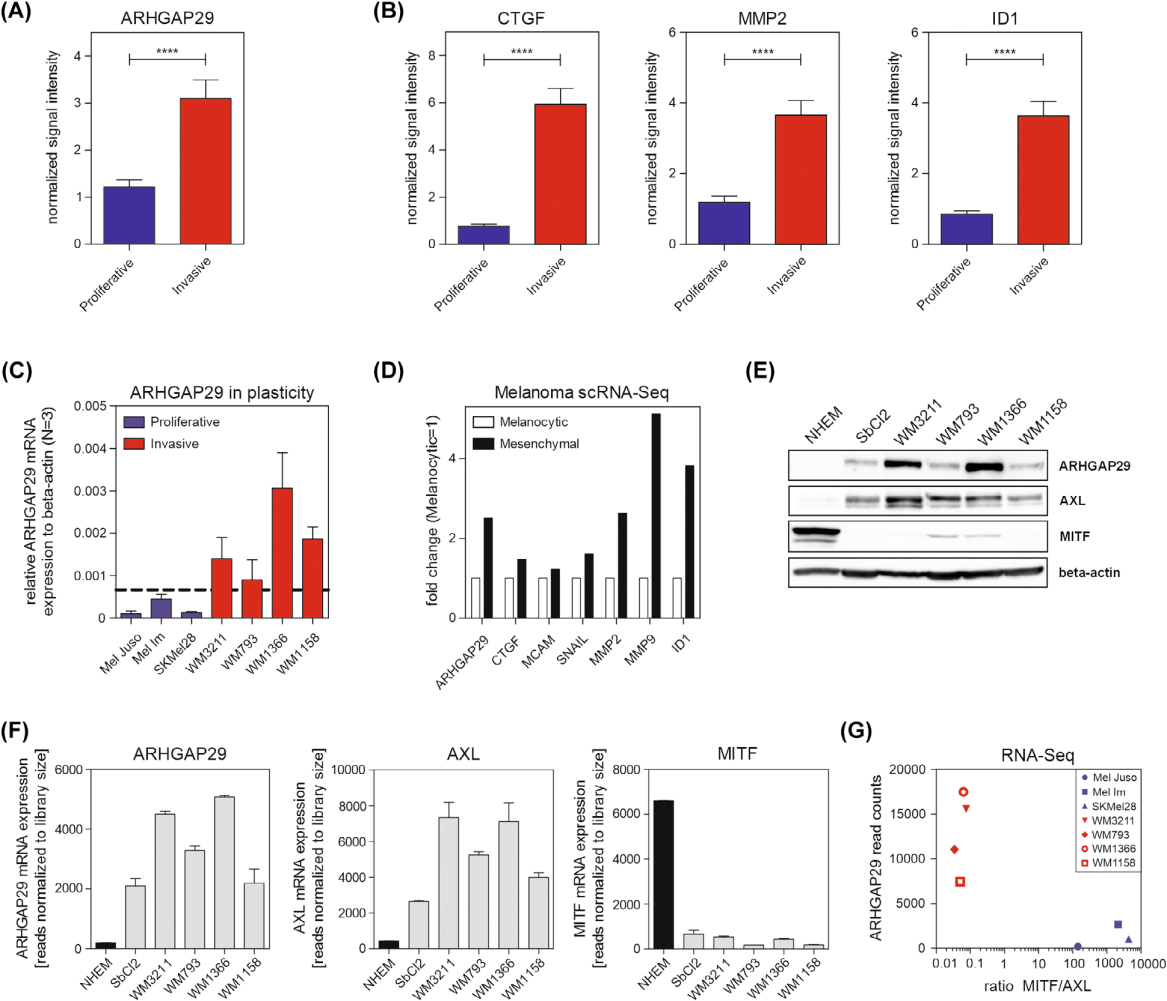
Undifferentiated, invasive melanoma cells display an increased expression of ARHGAP29. (A) Phenotype‐specific expression analysis of (A) ARHGAP29, (B) CTGF, MMP2, and ID1 in melanoma cell lines based on a published dataset of Widmer *et al*. [44]. (C) Relative mRNA expression level of ARHGAP29 in different melanoma cell lines categorized as proliferative (blue) and invasive (red) phenotypes according to Widmer *et al*. [44]. (D) Analysis of single‐cell RNA sequencing (scRNA‐Seq) data showing the expression of different genes in mesenchymal‐like melanoma cells compared to melanocytic melanoma cells (Melanocytic = 1). The used dataset derived from Pozniak *et al*. [45]. (E) Protein expression levels and (F) Analysis of RNA sequencing count data [25] of ARHGAP29, AXL, and MITF in different melanoma cell lines and NHEMs. (G) Analysis of RNA sequencing data [25] of different melanoma cell lines categorized according to ARHGAP29 read counts in dependence on the read counts of MITF/AXL ratio. Significance determined by Student's *t*‐test. Error bars depicting the mean ± SEM. ****P ≤ 0.0001.

Based on our observations that ARHGAP29 promotes cell invasion, activates SMAD signaling, and leads to a spread, mesenchymal‐like cell morphology, we finally hypothesized that ARHGAP29 may be involved in the switch from a proliferative to an invasive, undifferentiated cell state. Therefore, we used the platform described by Widmer *et al*. to investigate the melanoma phenotype‐specific expression of ARHGAP29 [44]. Indeed, the analysis revealed an increased expression of ARHGAP29 in invasive compared to proliferative cell lines (Fig. 8A), which was also shown for the expression of CTGF, MMP2, and ID1 (Fig. 8B). For a comparison with the *in silico* data, we expanded the number of cell lines and correlated the ARHGAP29 mRNA expression of the different cell lines with the phenotypic classification from Widmer *et al*. regarding proliferative (depicted in blue) versus invasive (red) phenotype (Fig. 8C). Interestingly, we found a correlation between an enhanced ARHGAP29 expression and an invasive phenotype. Furthermore, we utilized the dataset from Poźniak *et al*. to analyze the expression pattern of ARHGAP29 in different melanoma cell states [45]. Again, we discovered that ARHGAP29 is more strongly expressed in mesenchymal‐like compared to melanocytic melanoma cells and detected the same pattern for most of the genes that are regulated by ARHGAP29 (Fig. 8D). To confirm these data, we examined the protein expression of melanocyte‐inducing transcription factor (MITF) and AXL receptor tyrosine kinase (AXL) in melanoma cell lines and melanocytes (Fig. 8E). In agreement with the *in silico* data, we observed a high AXL and a low MITF expression in cell lines with a high ARHGAP29 expression. Finally, this finding was validated by our own RNA‐Seq data [25] depicting ARHGAP29, AXL, and MITF (Fig. 8F). To further investigate ARHGAP29's role in plasticity, we analyzed the reads of ARHGAP29 and the ratio of MITF/AXL reads in the cell lines shown in Fig. 8C. Again, this revealed a link between ARHGAP29 expression and plasticity (Fig. 8G). Altogether, our data suggest that ARHGAP29 drives the development of a mesenchymal‐like, invasive phenotype in malignant melanoma by influencing the morphology of the tumor cells and increasing the expression of genes involved in the phenotype switch.

## 4 Discussion

In this study, we investigated the so far unknown role of ARHGAP29 in malignant melanoma. We observed an ARHGAP29 upregulation in melanoma cell lines compared to melanocytes (Fig. 1A–D) and found that ARHGAP29 stimulates cell migration and invasion (Fig. 3A,B), which is in agreement with other cancer entities [17, 23, 46]. Additionally, we discovered a higher ARHGAP29 expression in invasive and mesenchymal‐like melanoma cells compared to melanocytic, proliferative melanoma cells (Fig. 8A,C–G), suggesting a role of ARHGAP29 in the phenotypic plasticity and intratumoral heterogeneity of melanoma. Supporting this finding, we discovered that ARHGAP29 leads to melanoma cell spreading by inhibiting ROCK (Fig. 2), promoting a morphology that resembles the mesenchymal‐like phenotype. While earlier studies have already shown the effect of the RhoA/ROCK pathway on cell morphology [47], our data highlight ARHGAP29 as an important upstream regulator of the RhoA/ROCK signaling pathway as described in uveal melanoma [19]. The inhibition of RhoA/ROCK signaling by ARHGAP29 and the consequent change in cell shape was mimicked in siARHGAP29 cells by treatment with Y‐27632 (Fig. 6A). This is supported by studies in gastric cancer showing that ARHGAP29 inhibits the RhoA/ROCK/LIMK/cofilin pathway, resulting in a destabilization of filamentous actin and leading to cytoskeletal rearrangements and an increase in cell migration [23]. Similar findings were made in prostate cancer cell lines [24]. ARHGAP29 therefore promotes the development of an invasive and metastatic phenotype in different types of cancers, which seems to be similar in melanoma [17, 18, 23, 24]. Therefore, we conclude that ARHGAP29 can promote the change in melanoma cell morphology by influencing the aforementioned signaling pathway, resulting in a mesenchymal, migratory phenotype with a flexible cytoskeleton and an increased invasive capacity.

Besides the changes in cell morphology, we demonstrated that ARHGAP29 regulates genes that support melanoma cell motility and promote metastasis formation. We observed a stimulating effect of ARHGAP29 on MCAM (Fig. 4C–E), which already has been demonstrated to promote the homotypic adhesion between melanoma cells and the heterotypic adhesion between melanoma and endothelium cells, consequently facilitating metastasis formation [48]. Additionally, we discovered an upregulation of SNAIL, ITGB3, and MMPs by ARHGAP29 (Fig. 5). Published data have shown that SNAIL is a driver of cell invasion and is involved in the regulation of MMP2 expression in melanoma [49, 50]. MMPs are essential for cell invasion as they remodel and degrade the ECM. Especially, MMP2 has been described as an important player in melanoma cell invasion and metastasis formation [41]. In concert with the stimulation of ITGB3 expression, which supports the remodeling of the ECM by MMPs [42], ARHGAP29's effects on gene expression are linked to increased cell motility (Fig. 3A,B). The regulation of SNAIL and MMPs seemed to be less profound in *WM1158* (Fig. 5A–E), matching the observation of a weaker effect on cell migration and invasion. These effects are likely affected by phenotypic plasticity and could be dependent on the ARHGAP29 expression level (Fig. 8C,G). Besides this, we found that ARHGAP29 increases CTGF expression in melanoma cells (Fig. 7B) and found a higher CTGF expression in invasive melanoma cell lines, suggesting a role in the phenotype switch (Fig. 8B). In agreement, earlier studies have shown that CTGF drives the invasive capacity of melanoma cells [28, 51].

Surprisingly, the regulation of gene expression by ARHGAP29 could not be entirely explained by its inhibitory influence on RhoA/ROCK, suggesting the involvement of other signaling cascades. We discovered that ARHGAP29 stimulates TGFβ activity (Fig. 7I) and SMAD2 activity partially independently of ROCK (Fig. 7C,G,H). It has been shown that the inhibition of SMAD signaling in melanoma reduces cell invasion [52]. Research on melanoma bone metastases has also demonstrated that SMAD signaling in melanoma increases the number of bone metastases in *in vivo* models [53]. Consistent with this, we detected a decreased cell invasion after ARHGAP29 knockdown (Fig. 3A,B) paralleled by reduced activation of SMAD signaling (Fig. 7A). Supporting our finding of an ARHGAP29 effect on SMAD activity, we observed a regulation of CTGF (Fig. 7B), which has been shown to be a SMAD target gene [54, 55]. In addition to this, MMP2, MMP9, MMP14, SNAIL, and MCAM have also been described to be regulated by SMAD signaling [56, 57, 58]. Therefore, it can be assumed that ARHGAP29 regulates gene expression by influencing SMAD activity. SMAD signaling has already been described to promote the phenotype switch in melanoma [59]. Hence, ARHGAP29 influences the actin cytoskeleton by inhibiting RhoA/ROCK and promotes the switch to an invasive gene expression signature by enhancing the SMAD signaling cascade.

The exact way that ARHGAP29 regulates the SMAD pathway still needs to be examined. However, as we did not discover drastic changes in the expression of different TGFβ family members, their receptors (Fig. S3C) nor in the BMP activity (Fig. S4A, B), there seems to be a TGF‐independent way of ARHGAP29 influencing SMAD2 activity. Studies have described an inhibitory influence of RhoB on the TGFβ signaling pathway. It has been reported that RhoB interacts with SMAD3 in different non‐melanoma cell lines, consequently preventing the phosphorylation of SMAD3 by TGFBR1 [60]. Whether RhoA might regulate SMAD signaling in a similar way in melanoma needs to be investigated. The activation of the SMAD pathway might also be a secondary effect of ARHGAP29, caused by the regulation of the expression of certain genes or the activation of involved signaling cascades. For instance, an effect on the inhibitors of TGFβ signaling SKI proto‐oncogene (SKI) and SKI like proto‐oncogene (SnoN) could affect SMAD activity [61]. Additionally, we observed an upregulation of ITGB3 by ARHGAP29 (Fig. 5F). Various studies have described the stimulating effect of ITGB3 on the activity of the SMAD signaling cascade [62, 63, 64]. The influence of ITGB3 on SMAD signaling is likely due to a physical interaction of ITGB3 with TGFBR2, which stimulates its tyrosine phosphorylation by SRC proto‐oncogene (SRC) [64, 65]. Additionally, while CTGF is a target gene of SMAD signaling, it has also been described to stimulate the TGFβ pathway [66], opening up the possibility of a positive feedback loop and adding to the complexity of this signaling network.

Interestingly, we observed that the inhibition of ROCK further increased some of the effects of the ARHGAP29 knockdown (Fig. S2; Fig. 7C,D,G,H), hinting at a possible interaction between the RhoA/ROCK pathway and the SMAD signaling cascade. Connections between the RhoA/ROCK pathway and the TGFβ signaling cascade have already been described in various studies. Rodríguez‐Vita *et al*. discovered an inhibiting influence of RhoA/ROCK on the TGFβ/SMAD pathway in vascular smooth muscle cells [67], whereas ROCK seems to lead to an increase of SMAD2 expression in lung fibroblasts [68]. In our study, an additive effect of the ROCK inhibitor and siARHGAP29 treatment on pSMAD2 intensity was demonstrated (Fig. 7G,H). This is supported by the already published data from Xu Ting *et al*. describing that the inhibition of ROCK leads to a decreased phosphorylation of SMAD2 [69]. Therefore, a crosstalk between the RhoA/ROCK and SMAD signaling pathways and a consequent regulation of SMAD activity by ROCK might also exist in melanoma cells.

Furthermore, while the ARHGAP29 knockdown seems to interfere with the mesenchymal‐like phenotype of melanoma cells and therefore affects the mesenchymal invasion strategy, we have not yet addressed the possibility that the tumor cells might instead utilize the amoeboid invasion strategy. The amoeboid‐like invasiveness is characterized by a rounded cell morphology and an enhanced contractility through a cortical actomyosin network that is driven by the Rho/ROCK signaling cascade [47, 70]. Furthermore, this type of movement does not require proteolytic ECM degradation and seems to be less dependent on cell‐matrix interactions [70]; therefore, a downregulation of MMPs and integrins may not have an impact on amoeboid invasiveness. Taking this into consideration, the ARHGAP29 knockdown may enhance the amoeboid invasion strategy due to the increased RhoA/ROCK activity. However, as a recent study has demonstrated the downregulation of ROCK in advanced melanoma [71], the mesenchymal invasion strategy might be favored. Furthermore, it was recently described that the SMAD2 signaling pathway could be involved in promoting the amoeboid state in melanoma. Thus, there is the possibility that ARHGAP29 might also affect the amoeboid state due to its effect on SMAD2 activity [72], and therefore, a knockdown of ARHGAP29 may target both invasion strategies. However, this will need to be further investigated. Nevertheless, the different modes of migration could implicate the requirement of a combinatorial treatment that inhibits both migration strategies in order to effectively abolish the migratory potential of melanoma cells.

Our data give new insights into ARHGAP29's functions in malignant melanoma and shed light on the signaling cascades that are affected by ARHGAP29. We showed that ARHGAP29 plays a role in developing an invasive cell phenotype, indicating a contribution to disease progression. An important reason for the aggressiveness of melanoma and the early formation of metastases is its phenotypic plasticity. The capacity of melanoma cells to switch their gene expression profile and take on different cellular states also causes the development of drug resistance [9]. Considering the findings of this study, ARHGAP29 could therefore be a promising novel target for the treatment of melanoma. Targeting ARHGAP29 could lead to the downregulation of various genes related to the drug resistance, mesenchymal‐like cell state. Whether this reverses the phenotype switch and increases the response of the tumor cells to immunotherapy or targeted therapy options will have to be addressed in further studies.

## 5 Conclusion

In summary, we investigated the so far undescribed role of ARHGAP29 in malignant melanoma. Our study highlights the influence of ARHGAP29 on cell morphology, number, and motility and demonstrates its influence on the expression of various tumor‐promoting genes and its involvement in the switch to an invasive, mesenchymal‐like phenotype. Additionally, our data provide first insights that ARHGAP29 not only signals through the RhoA/ROCK pathway, thereby affecting cell morphology, but also plays an important role in activating the SMAD signaling pathway in a partially ROCK‐independent manner.

## Conflict of interest

The authors declare that they have no competing interests.

## Author contributions

Study conceptualization and design: BCT, AKB, and NR; data collection: BCT, MK‐F, NR; analysis and interpretation of results: BCT, MK‐F, AKB, and NR; manuscript writing: BCT, MK‐F, AKB, and NR. All authors read and approved the published version of the manuscript.

## Supporting information


**Fig. S1.** Transient gene knockdown of ARHGAP29.
**Fig. S2.** ARHGAP29 affects gene expression independently of its inhibitory influence on ROCK.
**Fig. S3.** Analysis of the influence of ARHGAP29 on different signaling pathways.
**Fig. S4.** Analysis of the influence of ARHGAP29 on BMP signaling.
**Table S1.** Human melanoma cell lines used for this study.
**Table S2.** Primer sequences for qRT‐PCR.
**Table S3.** Antibodies for western blot analysis.

## Data Availability

The RNA‐Seq data [25] used in this study have been deposited in the NCBI BioProject database (https://www.ncbi.nlm.nih.gov/bioproject/) and can be accessed with the BioProject accession number PRJNA839865. The supplementary material of this article, Supplemental Figures and Tables, is available.
