# Supplementary material for: Emerging role of ARHGAP29 in melanoma cell phenotype switching
Source: Mol Oncol. 2025 Sep 4;20(2):348–68. doi: 10.1002/1878-0261.70114 (PMC12936434; doi:10.1002/1878-0261.70114)
Supplement: Supplementary file 1 — Fig. S1. Transient gene knockdown of ARHGAP29. Fig. S2. ARHGAP29 affects gene expression independently of its inhibitory influence on ROCK. Fig. S3. Analysis of the influence of ARHGAP29 on different signaling pathways. Fig. S4. Analysis of the influence of ARHGAP29 on BMP signaling. Table S1. Human melanoma cell lines used for this study. Table S2. Primer sequences for qRT‐PCR. Table S3. Antibodies for western blot analysis. [file MOL2-20-348-s001.pdf]

Supporting Information:

Emerging role of ARHGAP29 in melanoma cell phenotype switching

Authors

Beatrice Charlotte Tröster[1], Melanie Kappelmann-Fenzl[1,2], Anja Katrin Bosserhoff[1,3,4,*], Nicole Rachinger[1,*]

Affiliations

[1] Institute of Biochemistry, Friedrich-Alexander-University Erlangen-Nürnberg (FAU), 91054 Erlangen, Germany

[2] Faculty of Computer Science, Deggendorf Institute of Technology, Dieter-Görlitz-Platz 1, 94469 Deggendorf, Germany.

[3] Comprehensive Cancer Center Alliance WERA (CCC WERA), 91054 Erlangen, Germany

[4] Bavarian Cancer Research Center (BZKF), 91054 Erlangen, Germany

[*] Correspondence: Dr. Nicole Rachinger, Prof. Anja Katrin Bosserhoff,
Institute of Biochemistry
FAU Erlangen-Nürnberg
Fahrstraße 17
91054 Erlangen - Germany
Phone: +49 9131 85-24190
Email: anja.bosserhoff@fau.de; nicole.rachinger@fau.de

Tab. S1 Human melanoma cell lines used for this study

Human melanoma cell line	RRID	Source tissue	BRAF	NRAS
SbCl2	CVCL_D732	RGP melanoma	WT	p.Gln61Leu
WM3211	CVCL_6797	VGP melanoma	WT	WT
WM793	CVCL_8787	VGP melanoma	p.Val600Glu	WT
WM1366	CVCL_6789	VGP melanoma	WT	p.Gln61Leu
WM1158	CVCL_6785	MET, Lymph node	p.Val600Glu	WT
Mel Juso	CVCL_1403	PT	WT	Q61L
Mel Im	CVCL_3980	MET	V600E	WT
SKMel28	CVCL_0526	MET	V600E	WT

Primary tumor (PT): RGP = Radial growth phase, VGP = Vertical growth phase; MET = Metastatic; WT = wildtype

Tab. S2 Primer sequences for qRT-PCR

Primer	Forward Primer (5'-3')	Reverse Primer (5'-3')
ARHGAP29	ATATGGAGCCCACCCAAAGG	AGGGCCCCAGGAAACTAGAA
β-actin	CTACGTCGCCCTGGACTTCGAGC	GATGGAGCCGCCGATCCACACGG
BMP2	TGGATTCGTGGTGGAAGTGGC	AGGGCATTCTCCGTGGCAGTA
BMP4	GATTCCCGTCCAAGCTATC	TCCATGATTCTTGACAGCC
BMPR1A	GAGCATCTCAAGCAGACG	CCTGTACCTTTAATGTCTGCC
CTGF	CAGAACCACCACCCTGCCG	CGTACATCTTCCTGTAGTACA
ID1	GTATCTGCTTCGGGCTTCCA	TGATTCTTGGCGACTGGCT
ITGB3	ACACTGGCAAGGATGCAGTGAATT GTAC	CGTGATATTGGTGAAGGTAGACGTGG C
MCAM	TGTGAGGCAGAGAGTAGCCA	CCAGTGGTTGTGTTGGAGTCT
MMP2	GCTGGGAGCATGGCGATGGATACC	GGACAGAAGCCGTACTTGCCATCC
MMP9	CCGAGCTGACTCGACGGTGATGG	GAGGTGCCGGATGCCATTCACGTC
MMP14	GGAACCCTGTAGCTTTGTGTCTGT C	TCTCTACCCTCAACAAGATTAGATTCC
SNAIL	AGGCCCTGGCTGCTACAAG	ACATCTGAGTGGGTCTGGAG
TGFB1	GACTGCGGATCTCTGTGTCAT	AGTGCCCAAGGTGCTCAATAA
TGFB3	CCCTGACCATCCTGTACTATGTTGG G	GGGTAGCCCAAATCCCATTGCCACAC
TGFBR1	CCTTCCAAGATTCAACGTGGC	CCAGAGCAGCCTTCAGTCAA
TGFBR2	TTTGAGGACCAGTGTTCCCG	AGTAGATGGTGGGGCAATCG

Tab. S3 Antibodies for Western Blot analysis

Antibody	Dilution	Clone Information, Manufacturer
Anti-ARHGAP29	1:3 000	NBP1-05989, Novus Biologicals, Littleton, CO, USA
Anti-MCAM	1:3 000	ab75769, Abcam, Cambridge, UK
Anti-SNAIL	1:1 000	#3879, Cell Signaling Technology, Frankfurt, Germany
Anti-pSMAD2	1:1 000	#3108, Cell Signaling Technology, Frankfurt, Germany
Anti-pAKT	1:3 000	#4060, Cell Signaling Technology, Frankfurt, Germany
Anti-pERK	1: 3000	#4370, Cell Signaling Technology, Frankfurt, Germany
Anti-MITF	1:500	sc-515925, Santa Cruz Biotechnology, Dallas, TX, USA
Anti-AXL	1:1 000	#8661, Cell Signaling Technology, Frankfurt, Germany
Anti-β-actin	1:5 000	A5441, Sigma-Aldrich, Darmstadt, Germany
Anti-rabbit HRP	1:2 000	#7074, Cell Signaling Technology, Frankfurt, Germany
Anti-mouse HRP	1:2 000	#7076, Cell Signaling Technology, Frankfurt, Germany

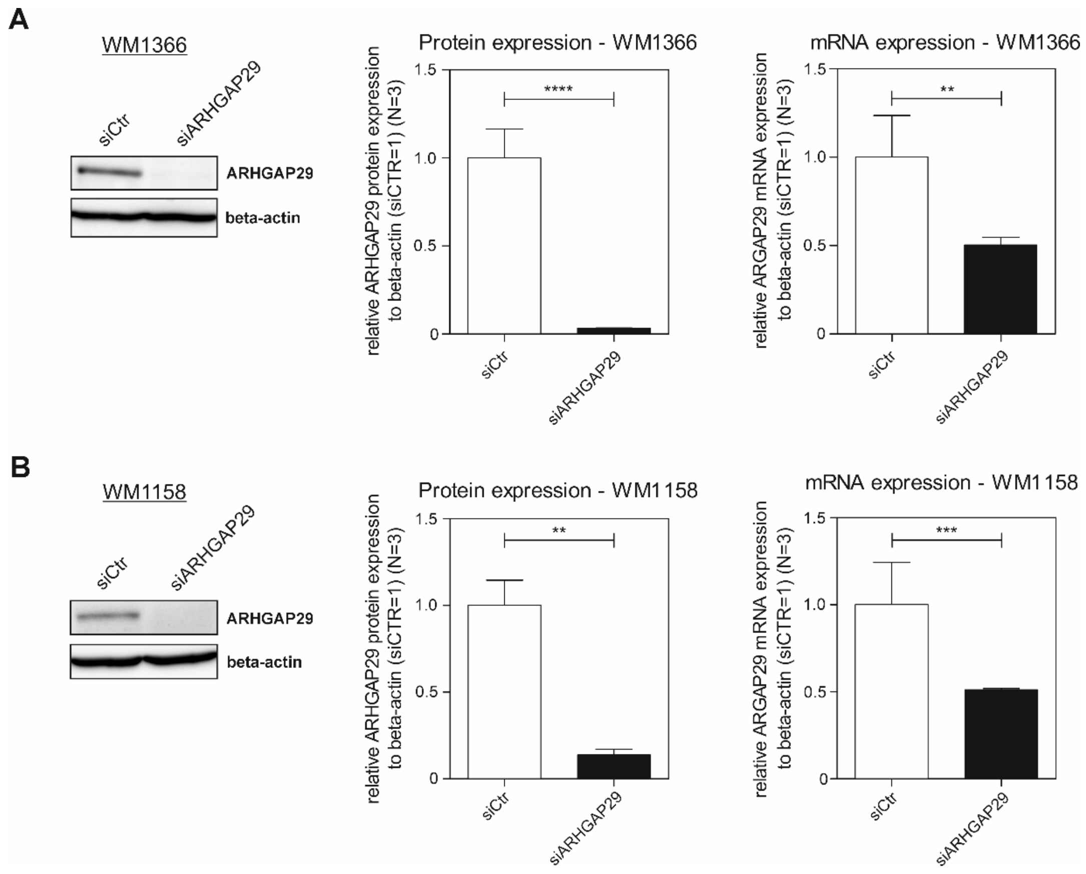

Fig. S1

Transient gene knockdown of ARHGAP29. Cells of the cell lines (A) *WM1366* and (B) *WM1158* were transfected with an siPool against ARHGAP29 (siARHGAP29) for 48 h. An siPool Control (siCtr) was used as a control (siCtr=1). The knockdown was confirmed by Western Blot and qRT-PCR (N=3). The expression of ARHGAP29 was normalized to β-actin. Significance determined by Student's *t*-test. Error bars depicting the mean ± SEM. **: $p < 0.01$, ***: $p < 0.001$, ****: $p \leq 0.0001$.

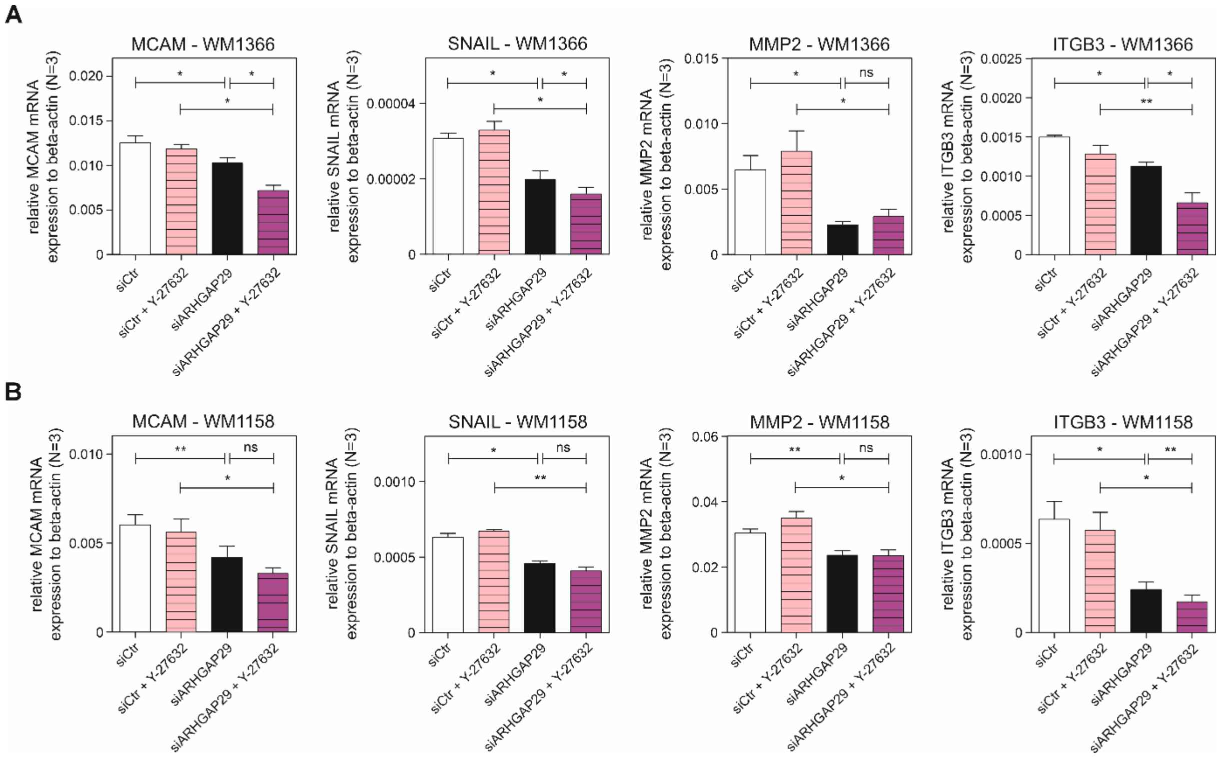

Fig. S2

ARHGAP29 affects gene expression independently of its inhibitory influence on ROCK. After the knockdown of ARHGAP29, the cells were treated with 10 µM of the ROCK inhibitor Y-27632. The expression of MCAM, SNAIL, MMP2 and ITGB3 was investigated in the cell lines (A) *WM1366* and (B) *WM1158* by qRT-PCR (N=3). The expression levels of the investigated genes were normalized to β-actin. Significance determined by Student's *t*-test. Error bars depicting the mean ± SEM. ns: not significant, *: $p < 0.05$, **: $p < 0.01$.

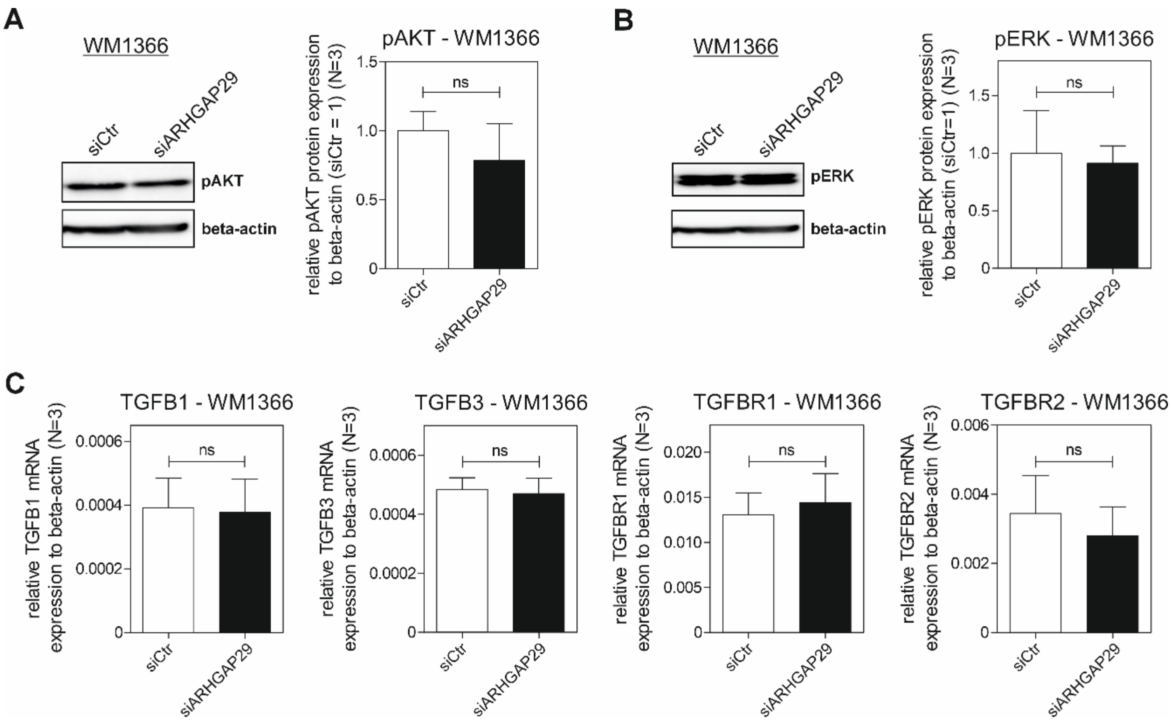

Fig. S3

Analysis of the influence of ARHGAP29 on different signaling pathways. The protein expression of (A) pAKT and (B) pERK was investigated in the cell line *WM1366* by Western Blot. The expression was normalized to β-actin (siCtr=1) (N=3). (C) The mRNA expression of different genes involved in the TGFβ signaling pathway was analyzed by qRT-PCR and the expression normalized to β-actin (N=3). Significance determined by Student's *t*-test. Error bars depicting the mean ± SEM. ns: not significant.

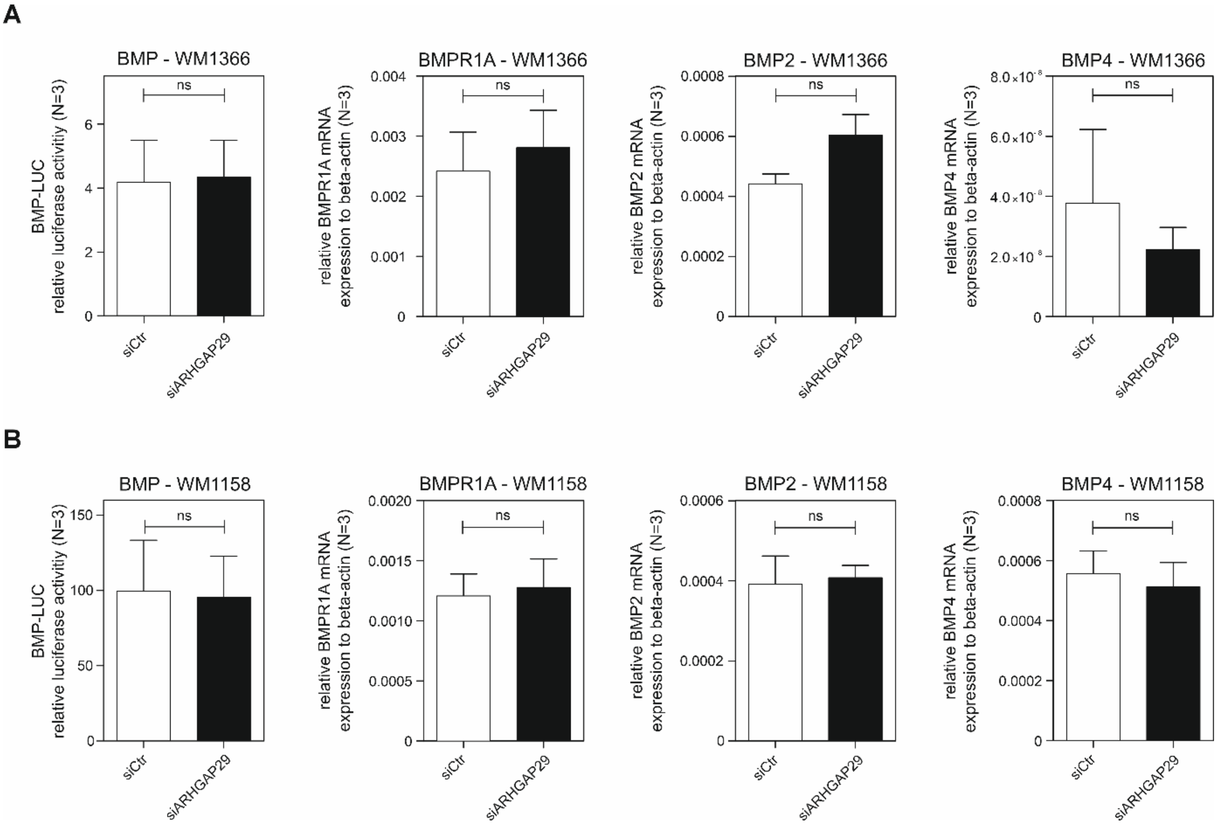

Fig. S4

Analysis of the influence of ARHGAP29 on BMP signaling. Relative luciferase activity in (A) *WM1366* and (B) *WM1158* cells transfected with a BMP-RE-Luciferase construct and relative mRNA expression level of BMPR1A, BMP2 and BMP4 normalized to β-actin in (A) *WM1366* and (B) *WM1158* (N=3). Significance determined by Student's *t*-test. Error bars depicting the mean ± SEM. ns: not significant.
